# Immunostimulatory activity and structure-activity relationship of epimedin B from *Epimedium brevicornu* Maxim.

**DOI:** 10.3389/fphar.2022.1015846

**Published:** 2022-10-31

**Authors:** Yuan Gao, Wei Shi, Can Tu, Peng Li, Guanyu Zhao, Xiaohe Xiao, Jiabo Wang, Zhaofang Bai

**Affiliations:** ^1^ School of Traditional Chinese Medicine, Capital Medical University, Beijing, China; ^2^ Senior Department of Hepatology, The Fifth Medical Center of PLA General Hospital, Beijing, China; ^3^ China Military Institute of Chinese Medicine, The Fifth Medical Center of PLA General Hospital, Beijing, China; ^4^ School of Life Sciences, Beijing University of Chinese Medicine, Beijing, China; ^5^ Beijing Research Institute of Chinese Medicine, Beijing University of Chinese Medicine, Beijing, China; ^6^ State Key Laboratory of Quality Research in Chinese Medicine, Institute of Chinese Medical Sciences, University of Macau, Macau, China

**Keywords:** epimedin B, idiosyncratic drug-induced liver injury, NLRP3 inflammasome, epimedii folium, mitochondrial ROS

## Abstract

Epimedii Folium (EF, *Epimedium brevicornu* Maxim.), a traditional botanical drug, is famous for treating bone fractures, joint diseases, and several chronic illnesses. However, some studies indicated that EF could induce idiosyncratic drug-induced liver injury (IDILI) in the clinic. The NLRP3 inflammasome plays a crucial role in the pathogenesis of various human diseases, including IDILI. In the present study, we showed that epimedin B could specifically facilitate nigericin- or ATP-induced NLRP3 inflammasome activation under synergistic induction of mitochondrial reactive oxygen species. Moreover, epimedin B resulted in activation of Caspase-1 and IL-1β secretion in a lipopolysaccharide (LPS)-mediated susceptibility mouse model. MCC950 pretreatment completely abrogated activation of the NLRP3 inflammasome and prevented liver injury. Importantly, several studies have confirmed that some active constituents of EF could enhance activation of the NLRP3 inflammasome and may be involved in the pathogenesis of EF-IDILI. No reports are available on whether the structure-activity relationship associated with the immunostimulatory activity in EF contributes to the pathogenesis of EF-IDILI. These findings have changed our conventional understanding about the more glycogen, the more immunostimulatory activity.

## Introduction

Idiosyncratic drug-induced liver injury (IDILI) is an uncommon but challenging clinical problem with respect to both diagnosis and management (Fontana, 2014; Hoofnagle and Björnsson, 2019; Uetrecht, 2019b; a). IDILI is unpredictable, not dose-dependent, and cannot be easily established in animal models (Benesic et al., 2018). Recently, with the widespread use of herbal and dietary supplements (HDS) worldwide, traditional Chinese medicine (TCM) and dietary supplements have gained prominence as the leading causes of IDILI (Bernal and Wendon, 2013; Shen et al., 2019). Specifically, the incidence of IDILI induced by HDS, such as Psoraleae Fructus, Polygoni Multiflori Radix and Epimedii Folium has increased in recent years (Wang et al., 2014; Lin et al., 2015; Tu et al., 2019; Gao et al., 2020). However, the precise pathogenesis remains elusive.

The NOD-like receptor family, pyrin domain containing 3 (NLRP3) inflammasome is a multiprotein complex that can orchestrate innate immune responses to pathogen-associated molecular patterns (PAMPs) and danger-associated molecular patterns (DAMPs) (Schroder and Tschopp, 2010; Elliott and Sutterwala, 2015). Upon activation, it leads to the cleavage of pro-caspase-1, subsequently resulting in pyroptosis and the production of interleukin 1β (IL-1β) and IL-18. Aberrant activation of the NLRP3 inflammasome has been associated with many chronic and degenerative diseases, such as Alzheimer’s disease, osteoarthritis, type 2 diabetes, gout, atherosclerosis, and liver disease (Szabo and Csak, 2012; Wen et al., 2012; Wu et al., 2017). Moreover, activation of the NLRP3 inflammasome may be a critical mechanism underlying the development of IDILI(Szabo and Csak, 2012; Wree et al., 2014a; Wu et al., 2017).

As a well-known TCM, Epimedii Folium (EF, *Epimedium brevicornu* Maxim.) has a medicinal history of over a thousand years in China and other countries in Asia, HDS containing EF are widely used for treating bone fractures, joint diseases, several chronic illnesses, for delaying aging, etc. Nevertheless, EF and its preparations have garnered significant interest because they can induce liver injury (Wree et al., 2014a; Zhang et al., 2019; Zhong et al., 2019; Gao et al., 2020). In our previous study, we reported that Icariside I and Icariside II can enhance activation of the NLRP3 inflammasome and may be involved in the pathogenesis of EF-IDILI(Wang et al., 2020; Gao et al., 2021). The major active constituents of EF are flavonoids, and over 60 types of flavonoids have been identified, among which epimedin A, B, C, and icariin are considered major bioactive components that constitute more than 52% of the total number of flavonoids in EF. However, Icariside I and Icariside II, which both contain one glucoside have more immunostimulatory activity. Yet, no reports are available on whether the structure-activity relationship is associated with the immunostimulatory activity in EF and contributes to the pathogenesis of EF-IDILI. In the current study, we demonstrated that epimedin B induced IDILI by promoting activation of the NLRP3 inflammasome both *in vivo* and *in vitro*. These findings have changed our conventional understanding about “the more glycogen, the more immunostimulatory activity”.

## Materials and methods

### Mice

C57BL/6 female wild-type mice (6–8 weeks) were obtained from specific pathogen-free (SPF) Biotechnology Co., Ltd., (Beijing, China). NLRP3 knockout (NLRP3^−/−^) mice were obtained from the National Center of Biomedical Analysis (NCBA, Beijing, China) and supplied by Dr. Tao Li. All mice were maintained at a temperature of 22–24°C under a 12-h light/dark cycle with *ad libitum* access to food and water. In this study, all animal protocols were performed according to the guidelines for care and use of laboratory animals and approved by the Animal Ethics Committee of the Fifth Medical Centre, Chinese People’s Liberation Army General Hospital (animal ethics committee approval No. IACUC-2017-003).

### Cell culture

Bone marrow-derived macrophages (BMDMs) were isolated from marrow of the femoral bone of wild type (WT) or NLRP3^−/−^ female C57BL/6 mice (10-week-old). BMDMs were cultured in the Dulbecco’s modified Eagle medium (DMEM) supplemented with 10% fetal bovine serum (FBS), 1% penicillin/streptomycin (P/S), and 50 ng/ml murine macrophage colony-stimulating factor (M-CSF). THP-1 cells were cultured in RPMI 1640 medium supplemented with 10% FBS and 1% P/S. Cells were maintained in a humidified 5% (v/v) CO_2_ incubator at 37°C.

### Antibodies and reagents

Adenosine triphosphate (ATP), Nigericin, SiO2, poly (deoxyadenylic-thymidylic) acid sodium salt (poly (dA:dT)), polyinosinic: polycytidylic acid [poly (I:C)], Pam3CSK4, dimethyl sulfoxide (DMSO), and LPS (Escherichia coli, 055: B5) were purchased from Sigma-Aldrich (Munich, Germany). Epimedin A (110623–72–8, purity 99.0%), epimedin A1 (140147–77–9, purity 99.92%), epimedin B (110623–73–9, purity 99.39%), epimedin C (110642–44–9, purity 99.1%), icariin (489–32–7, purity 97.64%), caritin (118525–40–9, purity 99%), and anhydroicaritin (38226–86–7, purity 99.51%) were purchased from TargetMol. *Salmonella* strains were kindly provided by Dr. Tao Li from the National Center of Biomedical Analysis. MCC950 was obtained from TargetMol (Boston, MA, United States). Anti-mouse caspase-1(1:1000, AG-20B-0042) was purchased from Adipogen (San Diego, CA, United States). Anti-mouse IL-1β (1:1000, 12,507) and anti-NLRP3 (1:2000, 15101S) antibodies were obtained from Cell Signaling Technology (Boston, MA, United States). Anti-ASC (1:1000, sc-22,514-R) was purchased from Santa Cruz Biotechnology (Dallas, TX, United States). Anti-GAPDH (1:2000, 60,004–1-1 g) was purchased from Proteintech (Chicago, IL, United States). The color prestained protein marker (20AB01) was purchased from GenStar (Beijing, China).

### Inflammasome activation

To induce activation of the inflammasome, BMDMs were seeded at a density of 5 × 10^5^ cells/well in 0.5 ml of medium in 24-well plates and were incubated overnight. The following day, medium was replaced with fresh medium, and BMDMs were subjected to stimulation with 50 ng/ml lipopolysaccharide (LPS) or 1 μg/ml Pam3CSK4 for a duration of 4 h. Next, cells were subjected to treatment with epimedin B for 1 h and were subsequently stimulated as follows: 5 mM ATP for 1 h, 7.5 μmol/L nigericin for 30 min, or 250 μg/ml silicon dioxide (SiO2) for 6 h. Cells were transfected with poly (I:C) (2 μg/ml), poly (dA:dT) (2 μg/ml), or LPS (1 μg/ml) for 6 h using Lipofectamine 2000 according to the manufacturer’s instructions.

### Caspase-1 activity assay

According to the manufacturer’s instructions (Promega, Madison, WI, United States), we used the 1:1 ratio of Caspase-Glo 1 Reagent volume/sample volume to assess caspase-1 activity in cell culture medium.

### Enzyme-linked immunosorbent assay

Mouse IL-1β (R&D Systems, Minneapolis, MN, United States), IL-1β, TNF-α, and IL-6 (Dakewe, Beijing, China), according to the manufacturer’s instructions, were used to measure the cell culture supernatants and mouse serum, respectively.

### Alanine aminotransferase and aspartate transaminase

Serum ALT and AST were determined using the commercially available assay kit (Nanjing Jiancheng Bioengineering Institute, Nanjing, China) according to the manufacturer’s instructions.

### Lactate dehydrogenase assay

The release of LDH into the culture supernatant was assessed using the CytoTox 96® 1 Non-radioactive Cytotoxicity Assay (Promega, Madison, WI, United States) according to the manufacturer’s instructions.

### ASC oligomerization

Cells were lysed with Triton buffer (50 mM Tris-HCl [pH 7.5], 150 mM NaCl, 0.5% Triton X-100, and EDTA-free protease inhibitor cocktail). The samples were then centrifuged at 6,000 × *g* at 4°C for 15 min. The supernatant was referred to as Triton X-soluble, and the pellet fractions were referred to as Triton X-insoluble fractions. To enable ASC oligomer cross-linking, the Triton X-100-insoluble fractions were subjected to washing steps and were resuspended in 200 μl of PBS, followed by the establishment of cross-linking at 37°C with 2 mM disuccinimidyl suberate (DSS) for 30 min. The pellets were centrifuged at 6,000 × *g* for 15 min, after which they were collected and dissolved in 1 × SDS loading buffer for immunoblot analysis.

### Intracellular K^+^ measurement

BMDMs were seeded in 12-well plates overnight and were primed with 50 ng/ml LPS for 4 h. The cells were subjected to treatment with epimedin B and were then stimulated with nigericin for 30 min. The culture medium was thoroughly aspirated and subjected to washing steps thrice using potassium-free buffer. Ultrapure HNO3 was added to perform lysis of the cells. Samples were collected in glass bottles and boiled for 30 min at 100°C. Intracellular K^+^ measurements were performed *via* inductively coupled plasma mass spectrometry.

### Measurement of intracellular Ca^2+^ levels

BMDMs were seeded at 2.5 × 10^4^ cells/mL overnight in a 384-well plate. After cells were primed with LPS for 4 h, they were treated with ATP for 45 min with or without epimedin B. Ca^2+^ flux measurements were performed by the FLIPRT Tetra system (Molecular Devices, San Jose, CA, United States).

### Mitochondrial reactive oxygen species assay

BMDMs were seeded at a density of 1 × 10^6^ cells/mL in culture dishes with a diameter of 100 mm and primed with LPS (50 ng/ml) for 4 h. Next, cells were transferred to a test tube, subjected to wash steps with Opti-MEM, and were stimulated as per the methods described previously. For measurements the release of mitochondrial reactive oxygen species (ROS), BMDMs were subjected to staining procedures using 4 μM MitoSOX for 20 min at 37°C, followed by two wash steps with HBSS, and assessment using flow cytometry. After completion of the staining and wash steps, flow cytometry was performed to measure mtROS levels.

### Assessment of the effects of LPS/epimedin B cotreatment-induced DILI *in vivo*

C57BL/6 mice (6-8-week-old females) were subjected to starvation for 24 h and were administered LPS (2 mg/kg) or saline vehicle *via* intravenous (i.v.). Tail vein administration. Following an observation period of 2 h, epimedin B (20 mg/kg, 40 mg/kg, and 80 mg/kg) or the vehicle was administered *via* intraperitoneal injection. Serum and a fraction of liver tissues were collected 6 h after epimedin B treatment. Serum ALT, AST, IL-1β, and TNF-α levels were measured. Histopathological analysis was performed *via* hematoxylin and eosin (H&E) staining. The number of F4/80-positive macrophages in the liver was determined.

In the second experiment, female C57BL/6 mice (6–8 weeks) were administered MCC950 (40 mg/kg) or saline vehicle through intraperitoneal injection. After 1 h, LPS (2 mg/kg) or saline vehicle was administered i.v. *via* tail vein. After 2 h, epimedin B (40 mg/kg) was administered *via* intraperitoneal injection. Mouse serum and a fraction of liver tissues were collected after 6 h. H&E staining was performed, and serum levels of IL-1*β*, TNF-*α*, ALT, and AST were determined.

### Statistical analyses

For statistical analysis, GraphPad Prism 7 (GraphPad Software) and Microsoft Excel were used. Data are presented as means ± SD and analyzed using a standard two-tailed unpaired Student’s *t*-test for single comparisons and one-way ANOVA for multiple comparisons. Differences with a *p* value <0.05 were considered to be significant. Statistical significance is presented as *p < 0.05, **p < 0.01, ***p < 0.001 vs the control; NS, not significant.

## Results

### Epimedin B accelerates NLRP3 inflammasome activation

**FIGURE 1:**
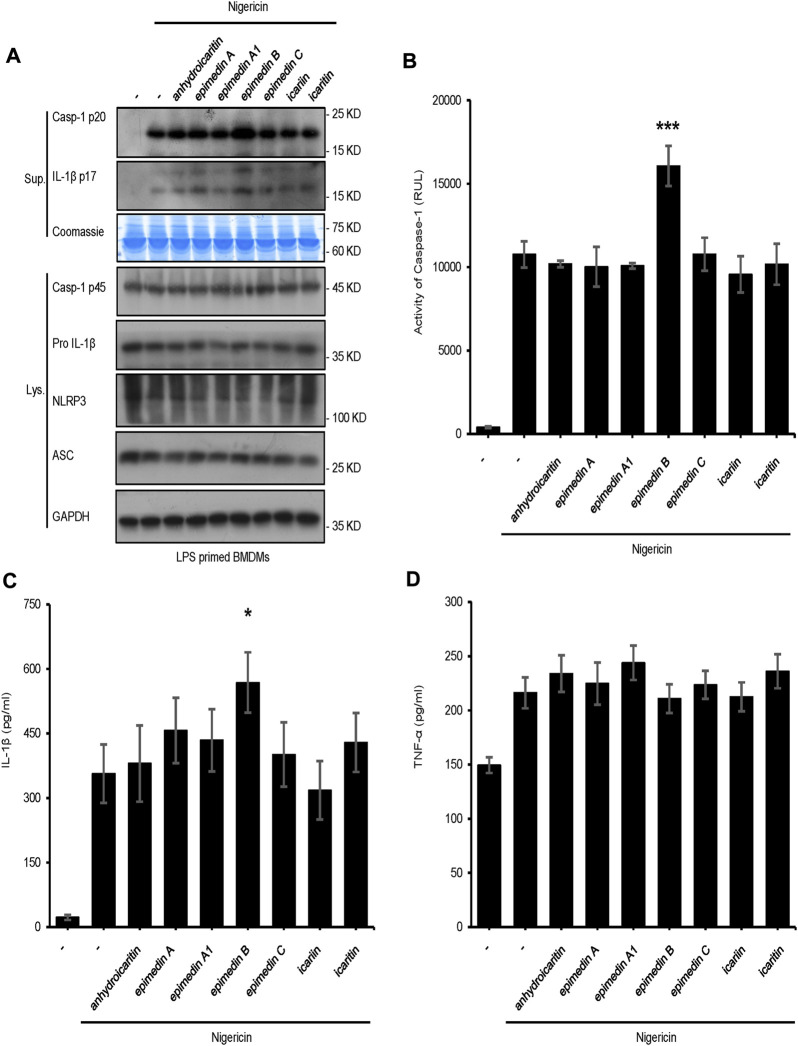
Effect of the main constituents of Epimedii folium on NLRP3 inflammasome activation. **(A)** Western blot analysis of supernatants (Sup) and whole-cell lysates (Lys) derived from lipopolysaccharide (LPS)-primed bone marrow-derived macrophages (BMDMs) subjected to treatment with the main constituents of EF (40 μM) and stimulated with nigericin (10 μmol/L). **(B–D)** Caspase-1 activity **(B)** ELISA of IL-1β **(C)**, and TNF-α **(D)** in Sup derived from the samples described in **(A)**. Data are expressed as the mean ± SD from at least three biological samples. The significance of the differences was analyzed using unpaired Student’s *t*-test: *p < 0.05, **p < 0.01, ***p < 0.001, NS; not significant, RLUs; relative light units.

**FIGURE 2:**
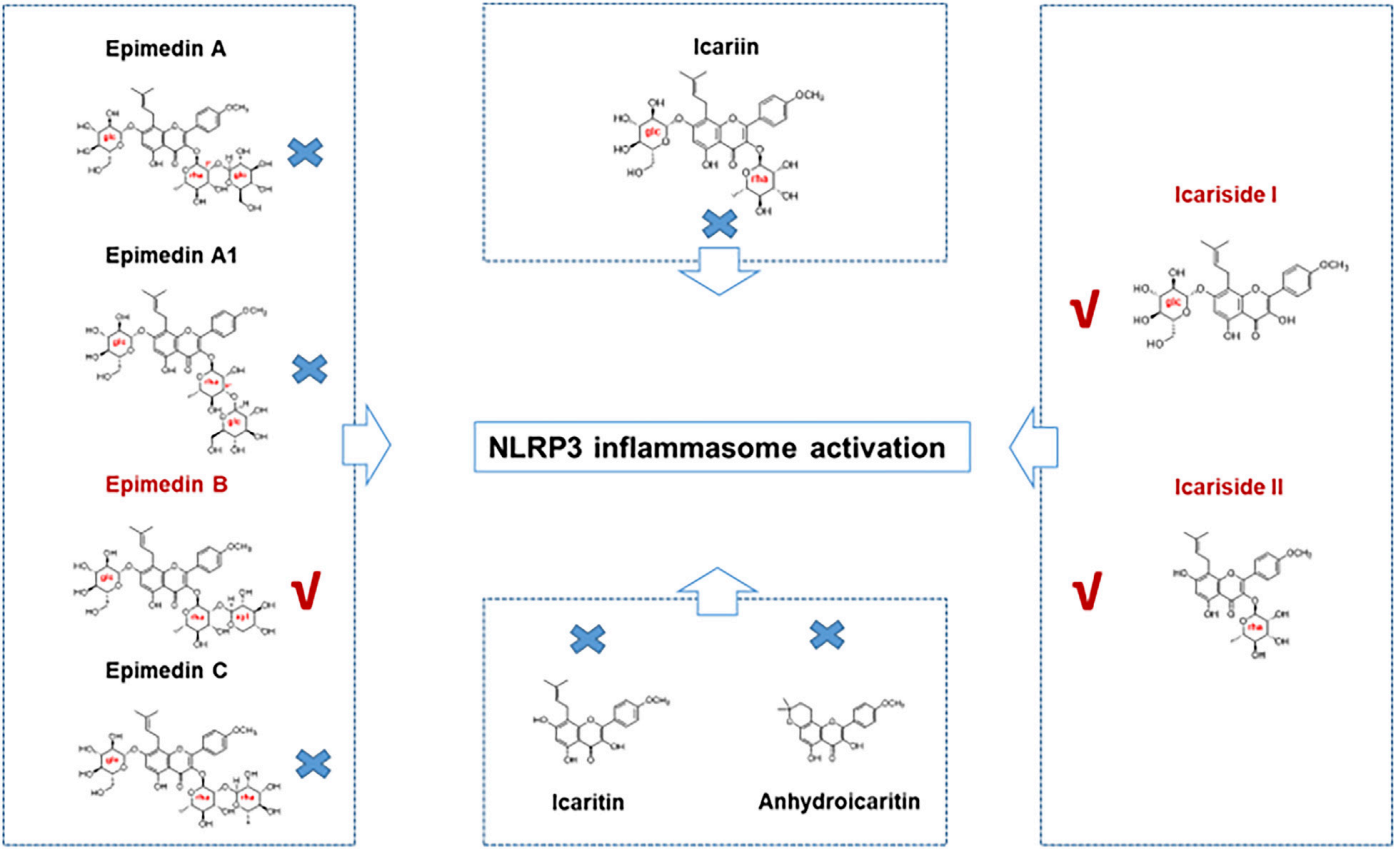
The relationship between the chemical structure and NLRP3 inflammasome activity of the main active constituents of Epimedii folium. Epimedin B, Icariside I, and Icariside II could promote NLRP3 inflammasome activation. Six other major active constituents of Epimedii folium (EF) (epimedin A, epimedin A1, epimedin C, icariin, icaritin, and anhydroicaritin) could not induce NLRP3 inflammasome activation.

**FIGURE 3:**
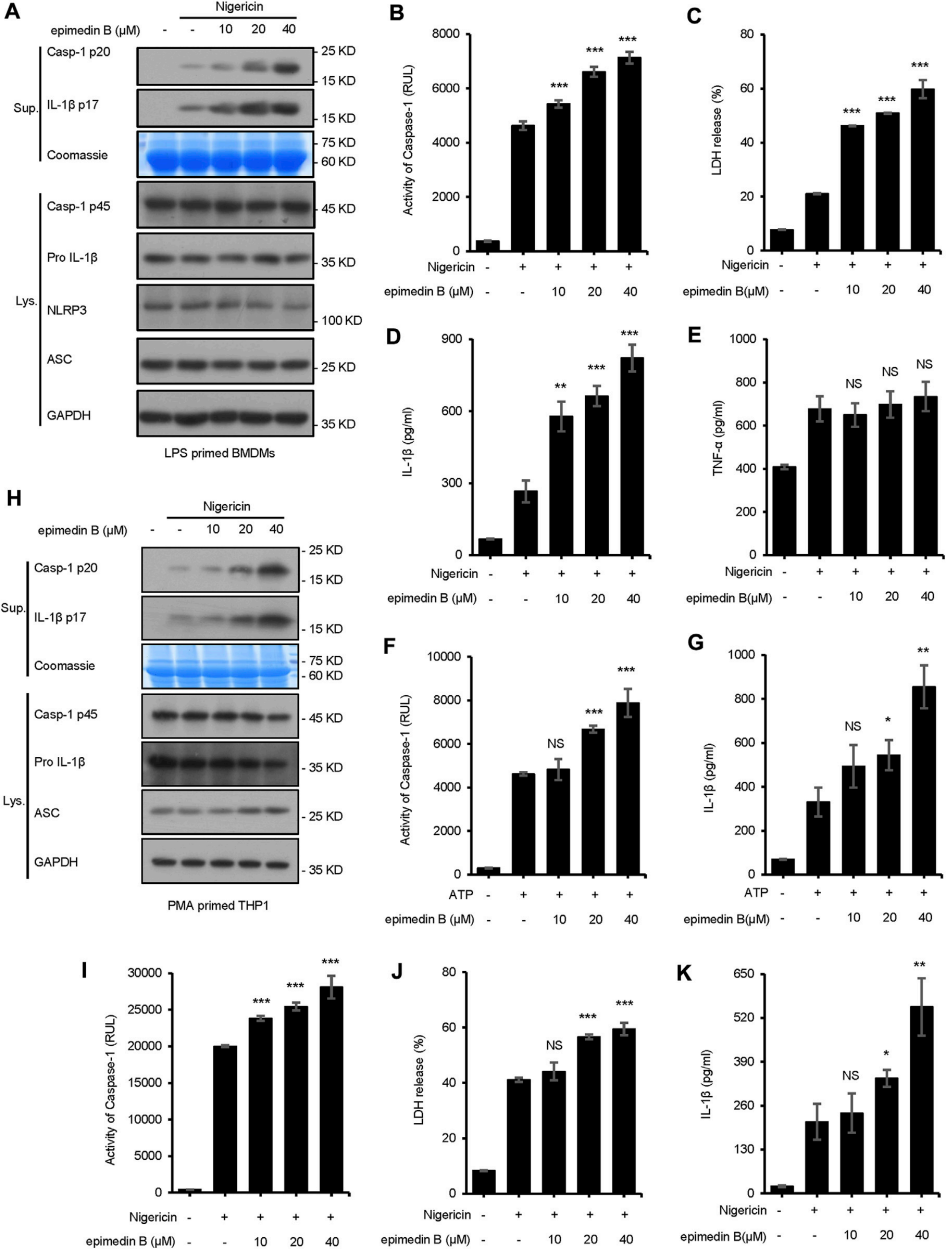
Epimedin B promotes NLRP3 inflammasome activation in bone marrow-derived macrophages and THP1 cells stimulated by nigericin or ATP. **(A)** Western blot analysis of supernatants (Sup) and whole-cell lysates (Lys) derived from lipopolysaccharide (LPS)-primed bone marrow-derived macrophages (BMDMs) subjected to treatment with various doses of epimedin B prior to nigericin stimulation. **(B–E)** Caspase-1 activity **(B)**, the release of lactate dehydrogenase (LDH) **(C)**, ELISA of IL-1β **(D)** and TNF-α **(E)** levels in supernatants (Sup) from samples described in **(A) (F,G)** Caspase-1 activity **(F)** and ELISA of IL-1β **(G)** of Sup and Lys derived from LPS-primed BMDMs subjected to treatment with various doses of epimedin B prior to ATP stimulation. **(H)** Western blot analysis of Sup and Lys derived from PMA-primed THP1 cells subjected to treatment with various doses of epimedin B prior to nigericin stimulation. **(I–K)** Caspase-1 activity **(I)**, release of LDH **(J)**, and ELISA of IL-1β **(K)** in Sup from samples described in **(H)**. Data are presented as the mean ± SD from at least three biological samples. The significance of the differences was analyzed using unpaired Student’s *t*-test: *p < 0.05, **p < 0.01, ***p < 0.001, NS; not significant, RLUs; the relative light units.

In the present study, seven major active constituents of EF were analyzed for their ability to activate the NLRP3 inflammasome. Figures 1A–C shows that epimedin B significantly promoted the activation of caspase-1 and IL-1β production induced by nigericin, but did not affect the production of TNF-α (Figure 1D). Therefore, epimedin B enhanced nigericin-induced NLRP3 inflammasome activation. The relationship between chemical structure and NLRP3 inflammasome activity of the main active constituents of EF is summarized. Figure 2 shows that the amount of glycogen contained in the main active constituents of EF is independent on NLRP3 inflammasome activity. These findings have changed our conventional understanding about “the more glycogen, the more immunostimulatory activity. ”To determine whether epimedin B could accelerate NLRP3 inflammasome activation, caspase-1 activation and IL-1β secretion were measured. Epimedin B exhibited dose-dependent active effects on caspase-1 cleavage and IL-1β secretion (Figures 3A,B,D). In addition, lactate dehydrogenase (LDH) induced by nigericin promoted LPS-primed BMDMs (Figure 3C). We also assessed the effect of epimedin B on ATP-induced NLRP3 inflammasome activation. Our data showed that LPS-induced caspase-1 activation and IL-1β secretion in BMDMs could be induced by epimedin B. However, no effects were observed on TNF-α production (Figure 3E). Next, we also assessed the impact of epimedin B on ATP-induced NLRP3 inflammasome activation in BMDMs. The results showed that epimedin B treatment increased production of the caspase-1 and IL-1β and the release of LDH triggered by ATP (Figures 3F,G, Supplementary Figures. S1A–C). Furthermore, THP-1 cells were selected to measure the effect of epimedin B on nigericin-induced NLRP3 inflammasome activation. The results indicated that epimedin B enhanced caspase-1 maturation, IL-1β secretion, and LDH release in a dose-dependent manner in response to nigericin in PMA-primed THP-1 cells (Figures 3H–K).

Pretreatment with epimedin B promoted nigericin-induced caspase-1 cleavage and IL-1β release in WT BMDMs but not in NLRP3^−/−^ BMDMs (Supplementary Figure 3A). MCC950 is a small-molecule inhibitor of the NLRP3 inflammasome (Coll et al., 2019). In this study, it was evaluated whether activation of the NLRP3 inflammasome induced by epimedin B could be inhibited by treatment with MCC950. Our results indicated that epimedin B accelerated NLRP3 inflammasome activation, which could be inhibited by MCC950 (Supplementary Figure S3B).

**FIGURE 4:**
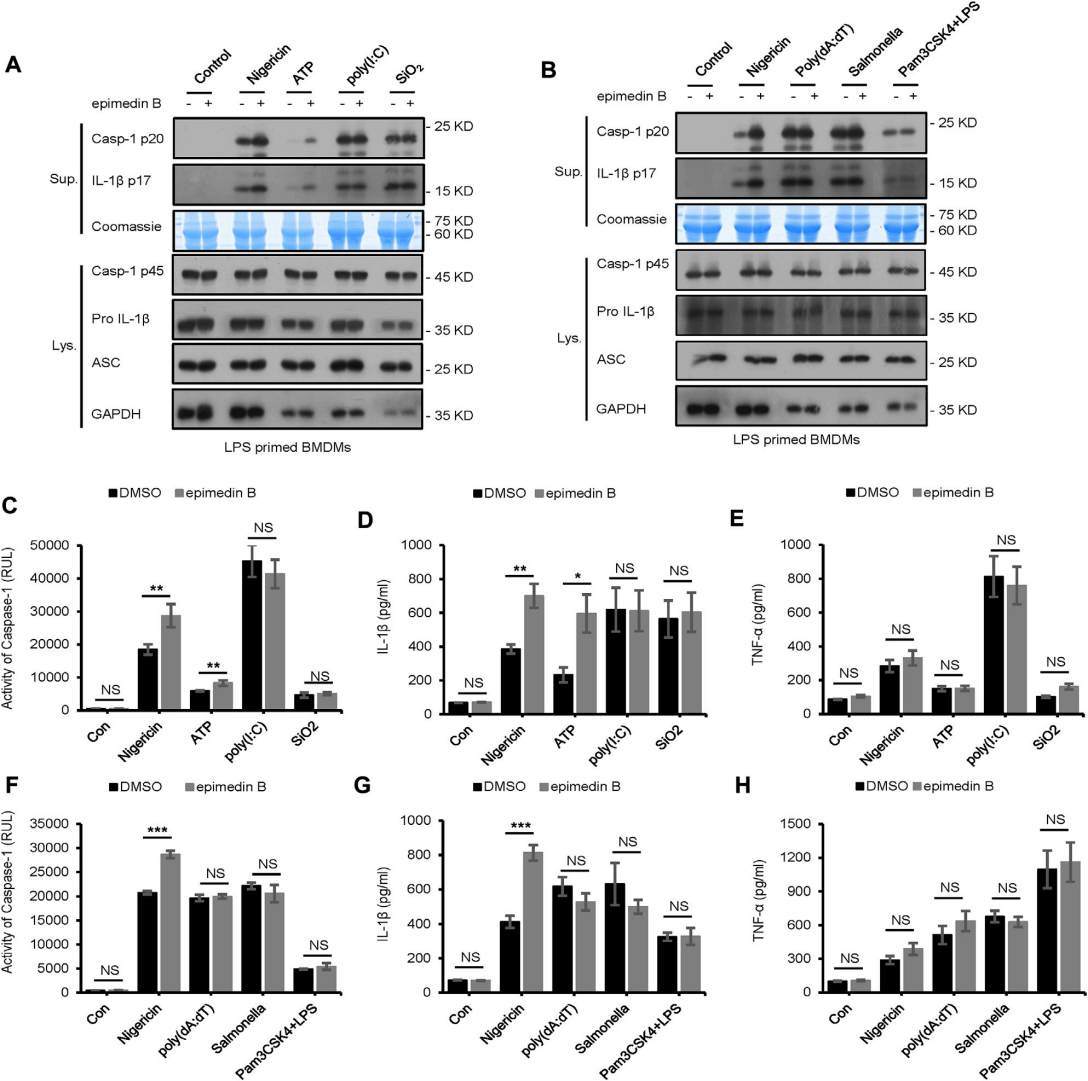
Epimedin B does not affect NLRP3 inflammasome activation induced by SiO2, poly (I:C), and intracellular lipopolysaccharide, as well as AIM2 and NLRC4 inflammasome. **(A,B)** Western blot analysis of supernatants (Sup) and whole-cell lysates (Lys) derived from lipopolysaccharide (LPS)/Pam3CSK4-primed bone marrow-derived macrophages (BMDMs) subjected to treatment with epimedin B and stimulated with nigericin, ATP, SiO2, poly (I:C), poly (dA:dT), Salmonella, or intracellular LPS. **(C–H)** Caspase-1 activity **(C,F)**, ELISA of IL-1β **(D,G)**, and TNF-α **(E,H)** in Sup derived from samples described in A and **(B)**. RLUs, relative light units. Data are presented as the mean ± SD from at least three biological samples. The significance of the differences was analyzed using unpaired Student’s *t*-test: *p < 0.05, **p < 0.01, ***p < 0.001, NS: not significant.

Moreover, we explored the effect of epimedin B on NLRP3 inflammasome activation initiated in response to other stimuli. Unexpectedly, treatment with epimedin B exerted no effect on caspase-1 cleavage and IL-1β secretion stimulated by other NLRP3 agonists, including SiO2 and poly (I:C) (Figures 4A, C–E). Furthermore, epimedin B did not affect cytosolic LPS, NLRC4, or AIM2 inflammasome activation (Figures 4B, F–H). Thus, these results indicate that epimedin B is a specific promoter that increases nigericin- and ATP-induced NLRP3 inflammasome activation.

**FIGURE 5:**
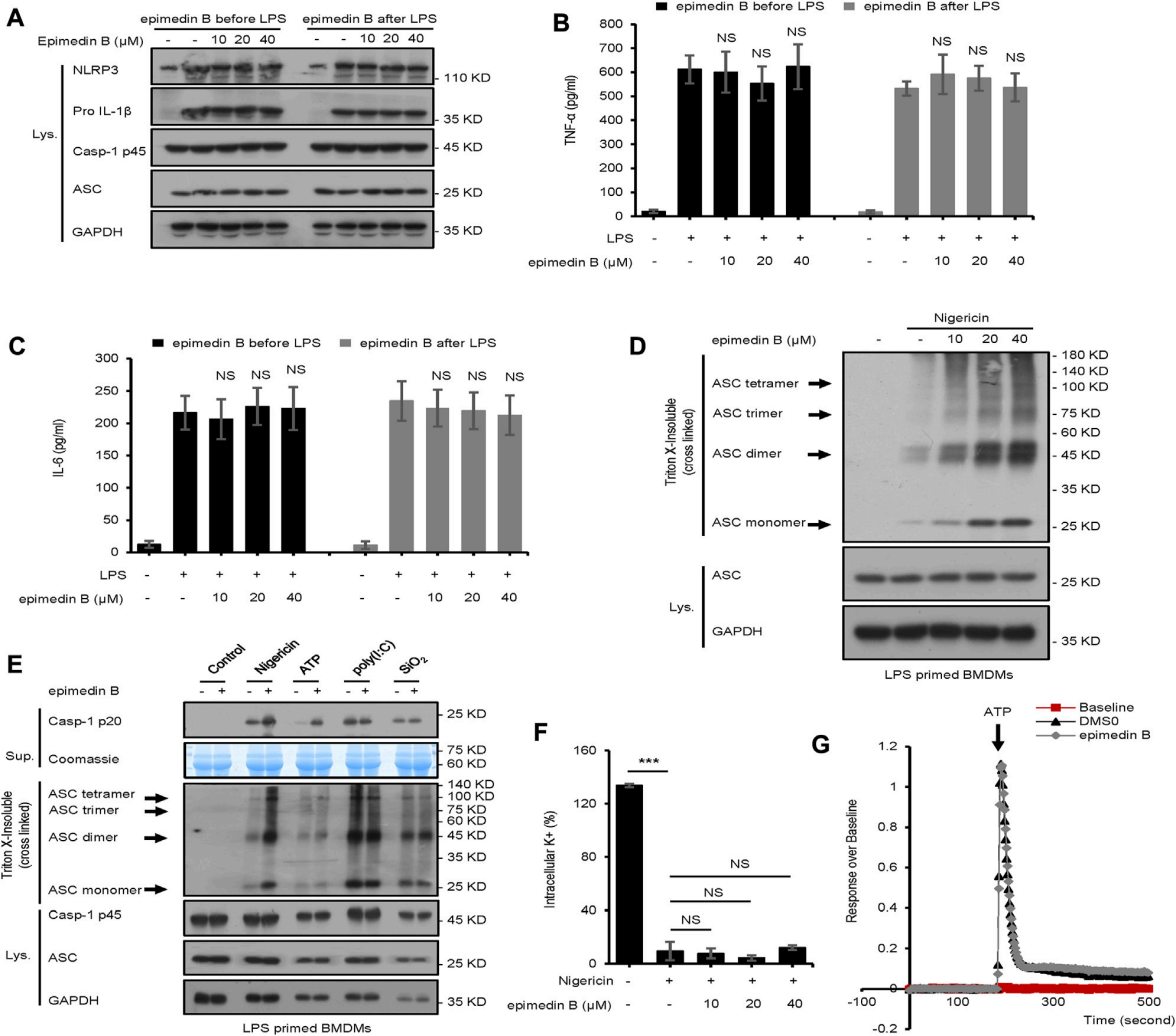
Epimedin B promotes ATP or nigericin-induced ASC oligomerization but does not block K^+^ efflux and Ca^2+^ flux. **(A)** Western blot analysis of whole-cell lysates from bone marrow-derived macrophages (BMDMs) subjected to treatment with epimedin B for 1 h, and stimulated with lipopolysaccharide (LPS) (50 ng/ml) for 3 h or BMDMs were stimulated with LPS (50 ng/ml) for 3 h and then subjected to treatment with epimedin B for 1 h **(B,C)** ELISA of TNF-α **(B)** and IL-6 **(C)** in Sup derived from samples described in **(A) (D)** Western blot analysis of ASC oligomerization from LPS-primed BMDMs subjected to treatment with various doses of epimedin B prior to nigericin stimulation. **(E)** Western blot analysis of ASC oligomerization from LPS-primed BMDMs subjected to treatment with epimedin B and stimulated with nigericin, ATP, SiO2, and poly (I:C). **(F)** Quantification of potassium efflux in LPS-primed BMDMs subjected to treatment with various doses of epimedin B and stimulated with nigericin. **(G)** A trace of ATP-induced Ca^2+^ flux was measured using the FLIPRTETRA system in LPS-primed BMDMs subjected to treatment with epimedin **(B)**. Data are presented as the mean ± SD from at least three biological samples. The significance of the differences was analyzed using unpaired Student’s *t*-test: *p < 0.05, **p < 0.01, ***p < 0.001, NS: not significant.

Next, we examined whether epimedin B affected LPS-induced priming for inflammasome activation. When BMDMs were stimulated with epimedin B before or after LPS treatment, epimedin B did not activate LPS-induced NLRP3 expression, or IL-6 and TNF-α production. The data presented in Figures 5A–C suggest that epimedin B does not enhance LPS-induced priming at the doses that are effective for NLRP3 activation, thereby suggesting that epimedin B exerts a robust effect on NLRP3 inflammasome activation.

### Epimedin B promotes nigericin or ATP-induced ASC oligomerization but does not block K^+^ efflux and Ca^2+^ flux

In this study, the mechanism underlying the activation of NLRP3 by epimedin B was investigated. First, our studies showed that epimedin B could activate nigericin-induced ASC oligomerization (Figure 5D), which is an essential step for NLRP3 activation. These findings suggested that epimedin B acts upstream of ASC oligomerization to exacerbate nigericin-induced NLRP3 activation. Second, epimedin B promoted ASC oligomerization induced by ATP (Figure 5E). However, epimedin B had no effect on ASC oligomerization induced by SiO2, poly (I:C), poly (dA:dT), *Salmonella typhimurium*, or cytosolic LPS (Figure 5E; Supplementary Figure S2). These data also indicated that epimedin B acted upstream of ASC oligomerization, which exacerbated activation of ATP- or nigericin-induced NLRP3 inflammasome. We also investigated whether epimedin B affected K^+^ efflux during NLRP3 inflammasome activation. The results showed that epimedin B exhibited no effect on K^+^ efflux triggered by nigericin (Figure 5F). Moreover, Ca^2+^ flux is an extremely important event in the upstream signaling of NLRP3 inflammasome activation. Epimedin B did not block ATP-induced Ca^2+^ flux (Figure 5G). Thus, Ca^2+^ flux may not be responsible for the enhanced effect of epimedin B on ATP-induced NLRP3 inflammasome activation.

### Epimedin B facilitates NLRP3 inflammasome activation by increasing mitochondrial ROS production

**FIGURE 6:**
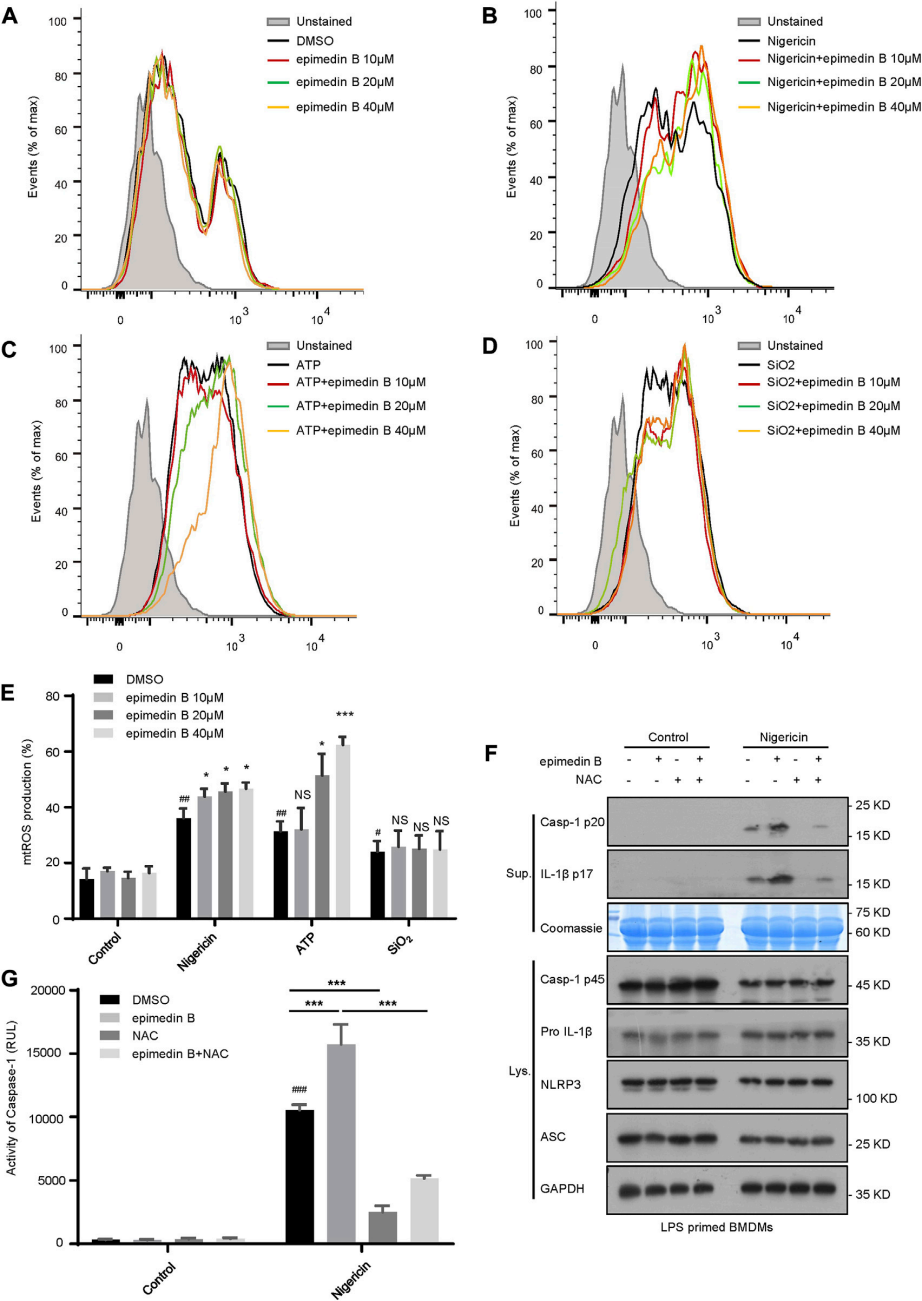
Epimedin B facilitates NLRP3 inflammasome activation by increasing mitochondrial reactive oxygen species (mtROS) production. **(A–D)** Percentage of ROS-positive cells in lipopolysaccharide (LPS)-primed bone marrow-derived macrophages (BMDMs) subjected to treatment with epimedin B that were either not stimulated **(A)** or stimulated with nigericin **(B)**, ATP **(C)** or SiO2 **(D)**, followed by staining with MitoSox. After completion of the staining and washing procedures, flow cytometry was conducted to determine the level of mtROS production. **(E)** Percentage of reactive oxygen species (ROS)-positive cells in LPS-primed BMDMs subjected to treatment with epimedin B that were either not stimulated or stimulated with nigericin, ATP, or SiO2. **(F)** Western blot analysis of supernatants and whole-cell lysates derived from LPS-primed BMDMs subjected to treatment with epimedin B, NAC, or epimedin B plus NAC prior to stimulation with nigericin or without stimulation. **(G)** Caspase-1 activity in samples described in **(F)**. Data are presented as the mean ± SD derived from at least three biological samples. The significance of the differences was analyzed using unpaired Student’s *t*-test: ^#^ *p* < 0.05, ^##^ *p* < 0.01, ^###^ *p* < 0.001, *p < 0.05, **p < 0.01, ***p < 0.001, NS: not significant.

Mitochondrial ROS play a crucial role in NLRP3 inflammasome activation (Zhao et al., 2019). In our study, we investigated whether epimedin B-mediated mitochondrial ROS was involved in NLRP3 inflammasome activation. Our data show that epimedin B successfully potentiated mitochondrial ROS production induced by nigericin and ATP but not by SiO2 (Figures 6A–E). We focused on the ROS scavenger N-acetylcysteine (NAC), which is an inhibitor of mitochondrial ROS production. Mitochondrial ROS production was suppressed by NAC treatment. As expected, when stimulated with nigericin, NAC treatment reversed epimedin B-induced caspase-1 maturation or IL-1β production (Figures 6F,G). Taken together, these results indicated that epimedin B increased mitochondrial ROS production to facilitate nigericin-induced NLRP3 inflammasome activation.

### Epimedin B induces the development of IDILI by promoting NLRP3 inflammasome activation *in vivo*

**FIGURE 7:**
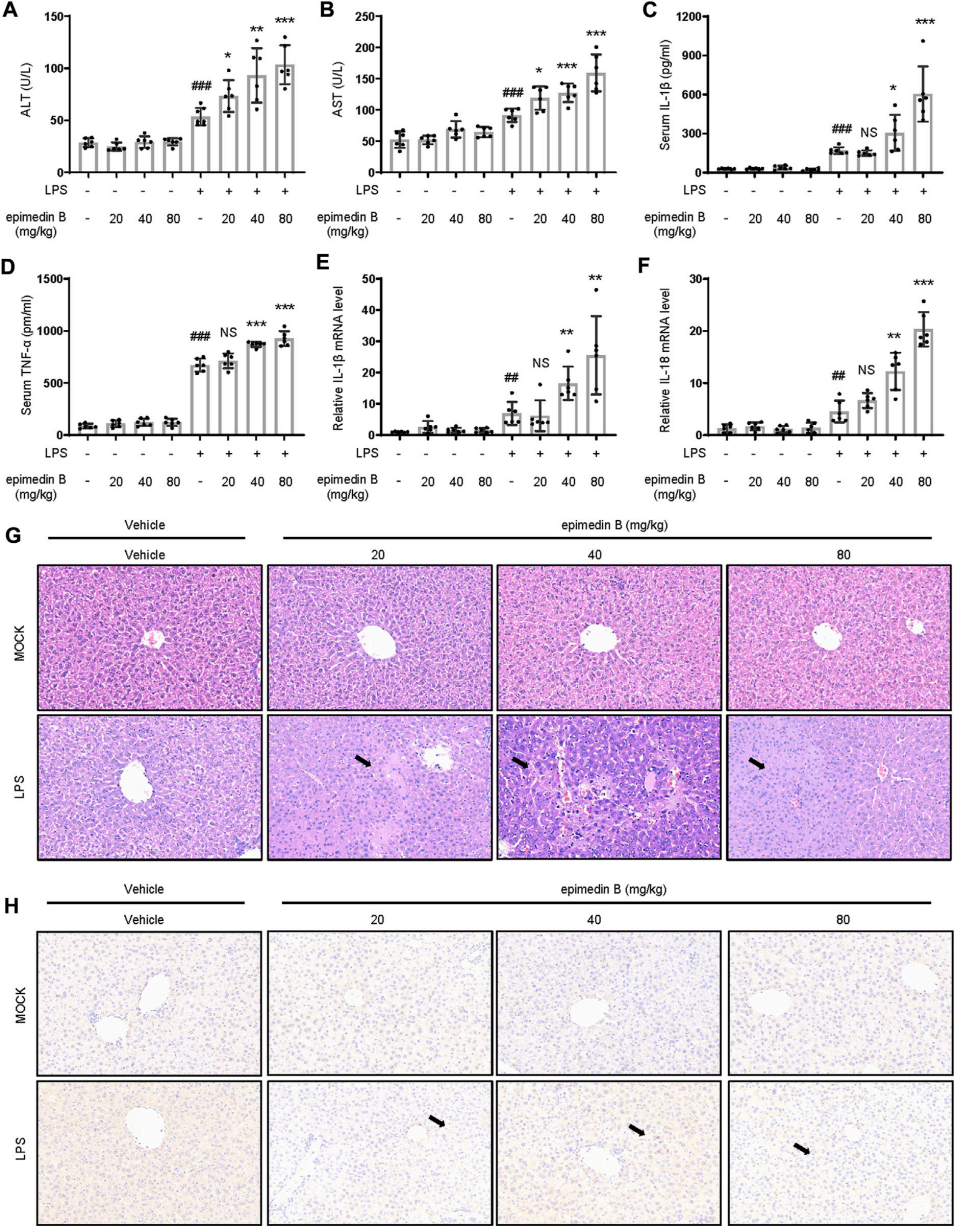
Epimedin B promotes early liver injury and inflammatory mediator production *in vivo*. **(A–H)** Female C57BL/6 mice (age: 6–8 weeks) subjected to starvation for 24 h were administered with 2 mg/kg of lipopolysaccharide (LPS) or its saline vehicle *via* the tail vein (i.v.). After an observation period of 2 h, various doses of epimedin B (20 mg/kg, 40 mg/kg, 80 mg/kg) or its vehicle were administered through intraperitoneal injection for 6 h **(A,B)** Serum levels of ALT **(A)** and AST **(B)** and **(C,D)** Serum levels of IL-1β **(C)** and TNF-α **(D)** determined by ELISA. **(E,F)** PCR of IL-1β **(E)** and IL-18 **(F)** mRNA levels. **(G,H)** Representative micrographs of H&E staining **(G)** and F4/80 staining **(H)**. Data are presented as the mean ± SD. The significance of the differences was analyzed using unpaired Student’s *t*-test: ^#^ *p* < 0.05, ^##^ *p* < 0.01, ^###^ *p* < 0.001, *p < 0.05, **p < 0.01, ***p < 0.001, NS: not significant.

We next examined whether epimedin B could induce the development of IDILI by promoting NLRP3 inflammasome activation *in vivo*. First, we investigated whether epimedin B, which can activate the NLRP3 inflammasome, could induce liver injury in an LPS-mediated susceptibility mouse model of IDILI. The results showed that treatment with epimedin B alone did not alter plasma levels of ALT and AST. As expected, in the LPS-mediated mouse model, epimedin B increased the levels of ALT and AST, and induced an increase in the production of IL-1β and TNF-α compared to mice in the LPS group (Figures 7A–D). Similar results were observed for the mRNA expression of the pro-inflammatory genes IL-1β and IL-18 (Figures 7E,F). Additionally, liver histology analysis showed that the combination of LPS and epimedin B treatment resulted in inflammatory cell infiltration or hepatocyte focal necrosis (Figure 7G). To further explore the effects of epimedin B on the immunological reaction in liver tissue, immunohistochemical (IHC) analysis of liver samples was performed. IHC staining of liver sections revealed that epimedin B increased the infiltration of F4/80-positive macrophages in the liver (Figure 7H). Furthermore, our results demonstrated that epimedin B could result in liver injury as shown in the LPS-induced mouse model.

**FIGURE 8:**
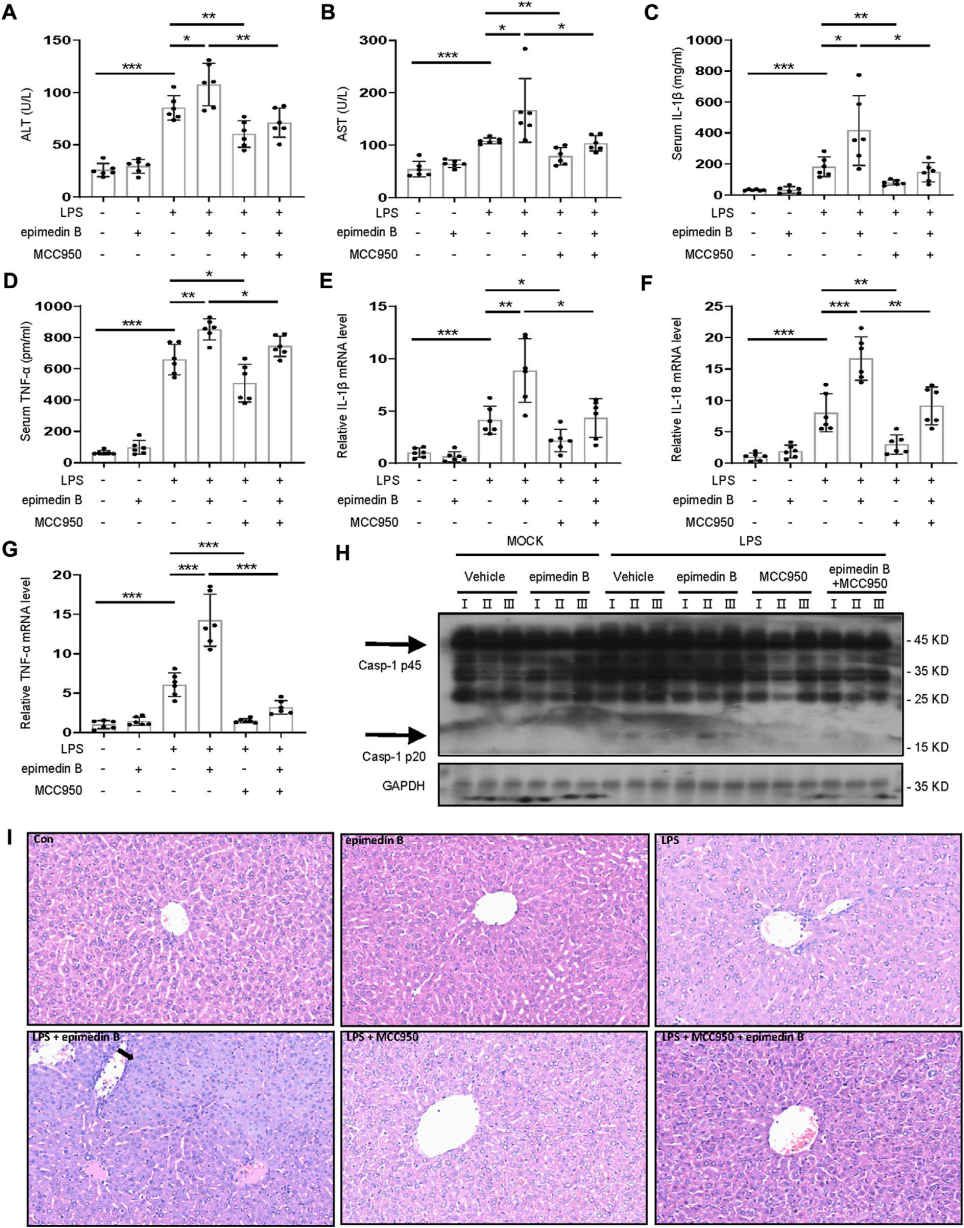
Epimedin B induces IDILI by promoting NLRP3 inflammasome activation *in vivo.* **(A–I)** Female C57BL/6 mice (age: 6–8 weeks) were administered MCC950 (40 mg/kg) or its saline vehicle through intraperitoneal injection. After 1 h, lipopolysaccharide (LPS) (2 mg/kg) or its saline vehicle was administered *via* the tail vein for 2 h. Subsequently, epimedin B (40 mg/kg) was administered *via* intraperitoneal injection for 6 h **(A,B)** Serum levels of ALT **(A)** and AST **(B)**, and **(C,D)** Serum levels of IL-1β **(C)** and TNF-α **(D)** determined by ELISA. **(E–G)** PCR of IL-1β **(E)**, IL-18 **(F)**, and TNF-α **(G)** mRNA levels. **(H)** Western blot analysis of pro-caspase-1 and cleaved caspase-1 expression in liver tissue. **(I)** Representative micrographs of H&E staining. Data are presented as the mean ± SD. The significance of the differences was analyzed using unpaired Student’s *t*-test: *p < 0.05, **p < 0.01, ***p < 0.001, NS: not significant.

MCC950 is a potent selective NLRP3 inhibitor (Coll et al., 2015). To verify the relationship between the NLRP3 inflammasome and epimedin B-induced liver injury, mice treated with MCC950 were selected. Figures 8A–D shows that the combination of LPS and epimedin B resulted in an increase in the levels of ALT, AST, IL-1β, and TNF-α. These results were not observed in mice co-treated with MCC950. Moreover, the mRNA expression of pro-inflammatory genes IL-1β, IL-18, and TNF-α was increased in the LPS group as well as in the group subjected to treatment with LPS in combination with epimedin B, but not in the MCC950 cotreatment group (Figures 8E–G). As shown in Figure 7H, MCC950 treatment suppressed caspase-1 activation in liver tissues of mice that were co-treated with epimedin B and LPS. Histology analysis of liver tissue showed that epimedin B treatment resulted in hepatocyte focal necrosis or inflammation in the LPS-mediated mouse model (Figure 8I). Thus, these results confirmed that epimedin B could activate the NLRP3 inflammasome, leading to liver injury *in vivo*.

## Discussion

IDILI represents a major global health issue. For instance, in North America, it has already surpassed viral hepatitis as a major factor of acute liver failure(Ostapowicz et al., 2002; Jee et al., 2021). However, in recent years, IDILI caused by TCM has been broadly recognized, especially in traditional non-toxic Chinese medicines. Thus, globally, there is a lack of understanding of possible IDILI mediated by TCM. Few traditional nontoxic Chinese medicines, such as EF, Psoraleae Fructus, and Polygoni Multiflori Radix have been reported to cause IDILI. Our previous studies have demonstrated that EF may induce hepatotoxicity in an LPS-mediated susceptibility mouse model of IDILI. Interestingly, in the present study, we demonstrated that epimedin B, which is one of the constituents of EF, specifically reinforced activation of the NLRP3 inflammasome to induce liver jury.

The NLRP3 inflammasome is one of the major contributors of inflammation and possesses the ability to sense both endogenous and exogenous danger signals through intracellular NLRs (Schroder and Tschopp, 2010; Elliott and Sutterwala, 2015). Several liver-related inflammatory diseases, especially chronic hepatitis C, nonalcoholic steatohepatitis, alcoholic liver disease, and IDILI have been reported to be associated with NLRP3 inflammasome activation (Csak et al., 2011; Burdette et al., 2012; Petrasek et al., 2012; Wree et al., 2014a; Wree et al., 2014b; Voican et al., 2015; Kato and Uetrecht, 2017). In the present study, we demonstrated that epimedin B facilitated nigericin- or ATP-induced NLRP3 inflammasome activation, thereby leading to the development of IDILI. Furthermore, epimedin B, *via* enhancement of NLRP3 inflammasome activation, contributes to EF-induced IDILI. In addition, the amount of glycogen contained in the main active constituents of EF is independent on NLRP3 inflammasome activity.

In the present study, epimedin B could only specifically reinforced activation of the NLRP3 inflammasome induced by nigericin or ATP. Additionally, epimedin B exerted no effect on the activation of AIM2 and NLRC4 inflammasomes. We showed that epimedin B specifically enhanced nigericin- or ATP-induced NLRP3 inflammasome activation. We also examined the effects of epimedin B on upstream and downstream signaling, and evaluated the mechanism underlying the enhancement of nigericin- or ATP-induced NLRP3 inflammasome activation by epimedin B treatment. ASC oligomerization is an important event in NLRP3 inflammasome activation. Epimedin B promoted ASC oligomerization triggered by nigericin and ATP. Therefore, we evaluated if epimedin B acted on the upstream signaling events of ASC oligomerization. K^+^ efflux or Ca^2+^ flux are deemed upstream mechanisms associated with NLRP3 inflammasome activation. However, our results demonstrated that epimedin B did not alter K^+^ efflux or Ca^2+^ flux. Moreover, mitochondrial damage and the release of mitochondrial ROS are key upstream events of NLRP3 inflammasome activation. The findings in this study showed that nigericin and ATP could induce the production of mitochondrial ROS. Notably, epimedin B specifically amplified the production of mitochondrial ROS triggered by nigericin and ATP, but not by SiO2, thus suggesting that epimedin B facilitated nigericin- or ATP-induced NLRP3 inflammasome activation dependent on mitochondrial ROS production. Next, we evaluated whether ROS played an important role in the enhanced effect of epimedin B on nigericin- or ATP-induced NLRP3 inflammasome activation. We concluded that the effect of epimedin B was dependent on mitochondrial ROS production for facilitating nigericin-induced NLRP3 inflammasome activation.

We previously reported that EF combined with non-hepatotoxic doses of LPS can induce liver injury. Therefore, we investigated whether epimedin B as an NLRP3 inflammasome activation promoter could cause liver injury. Our results indicated that epimedin B could induce liver injury *in vivo*. MCC950 was used to explore the relationship between epimedin B-induced liver injury and the NLRP3 inflammasome. The combination of epimedin B and LPS-induced liver injury but not in mice that received MCC950 pretreatment. Together, these data clearly demonstrated that epimedin B induced IDILI by promoting NLRP3 inflammasome activation *in vivo*.

## Conclusion

In conclusion, our study demonstrated that epimedin B induced NLRP3 inflammasome activation triggered by nigericin and ATP. Mitochondrial ROS are crucial contributors of the enhancement of the activation of the NLRP3 inflammasome stimulated by epimedin B. Treatment with a combination of nonhepatotoxic doses of LPS and epimedin B increased the production of ALT, AST, IL-1β, and TNF-α, resulting in hepatocyte necrosis. These results were not observed in mice that were co-treated with LPS and MCC950. Our findings indicated that epimedin B is responsible for EF-induced IDILI, and the amount of glycogen contained in the main active constituents of EF is independent on NLRP3 inflammasome activity.

## Data Availability

The original contributions presented in the study are included in the article/Supplementary Material, further inquiries can be directed to the corresponding authors.

## Abbreviations

IDILI, Idiosyncratic Drug-induced Liver Injury; TCM, Traditional Chinese Medicine; NLR, the Nucleotide-binding Domain and Leucine-rich Repeat; PAMPs, Pathogen-associated Molecular Patterns; IL-1β, Interleukin 1β; BMDMs, Bone Marrow-derived Macrophages; DMEM, Dulbecco’s Modified Eagle Medium; FBS, Fetal Bovine Serum; ATP, Adenosine Triphosphate; DMSO Dimethyl Sulfoxide; ELISA, Enzyme-linked Immunosorbent Assay; ALT, Alanine Aminotransferase; AST, Aspartate Transaminase; LDH, Lactate Dehydrogenase; ROS: Reactive Oxygen Species.
