# Supplementary material for: Immunostimulatory activity and structure-activity relationship of epimedin B from Epimedium brevicornu Maxim
Source: Front Pharmacol. 2022 Oct 31;13:1015846. doi: 10.3389/fphar.2022.1015846 (PMC9659593; doi:10.3389/fphar.2022.1015846)
Supplement: Supplementary file 1 [file DataSheet1.docx]

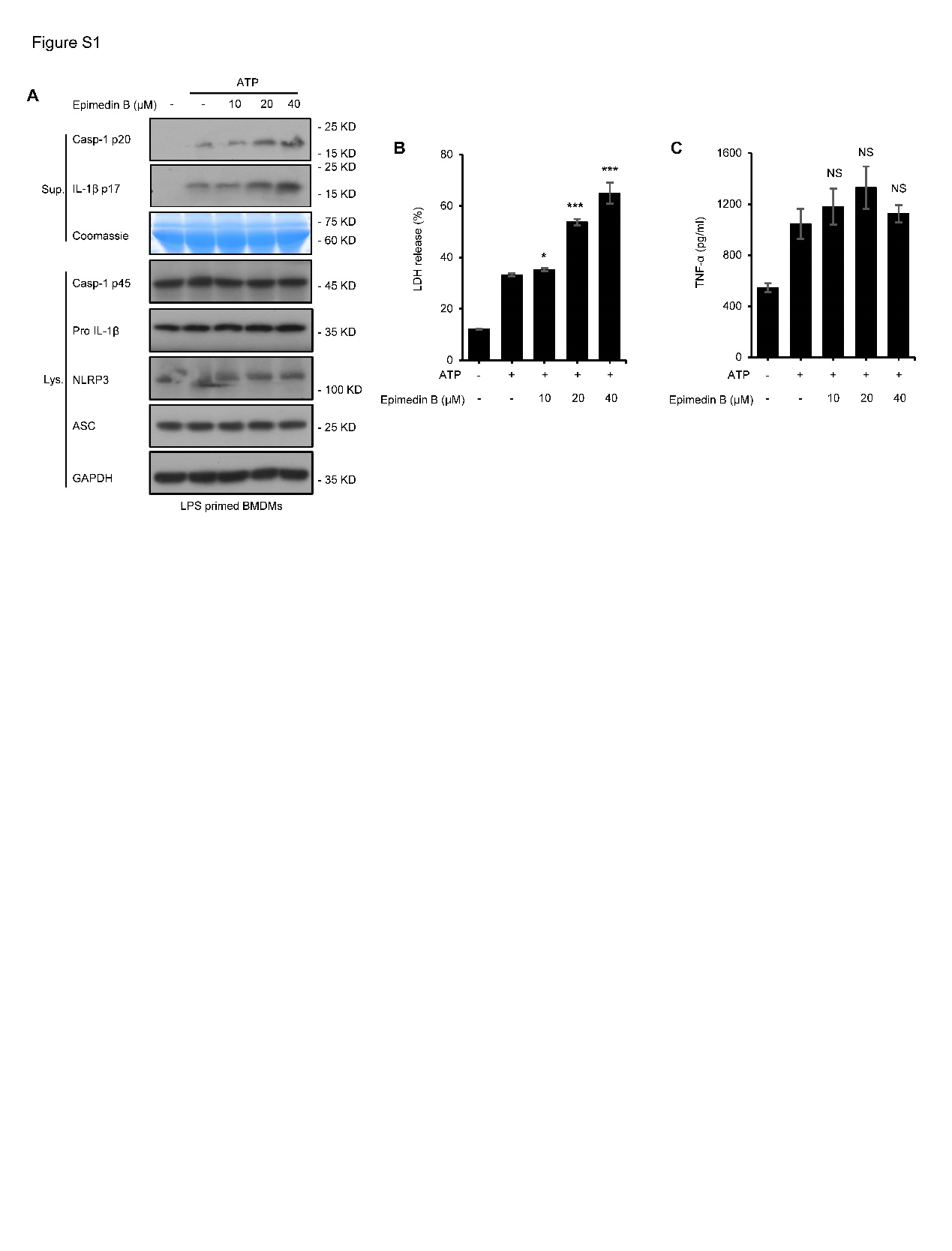


**Supplementary Fig 1 Epimedin B promotes NLRP3 inflammasome activation in BMDMs stimulated by ATP** (A) Western blot analysis of Sup. and Lys. derived from LPS-primed BMDMs subjected to treatment with various doses of epimedin B before ATP stimulation. (B-C) the release of LDH (B), ELISA of TNF-α (C) levels in supernatants (Sup) from samples described in A. Data are represented as mean ± SD from at least three biological samples. The significance of the differences was analyzed using unpaired Student’s *t*-test: *P <0.05, **P <0.01, ***P <0.001, NS; not significant, RLUs; the relative light units.


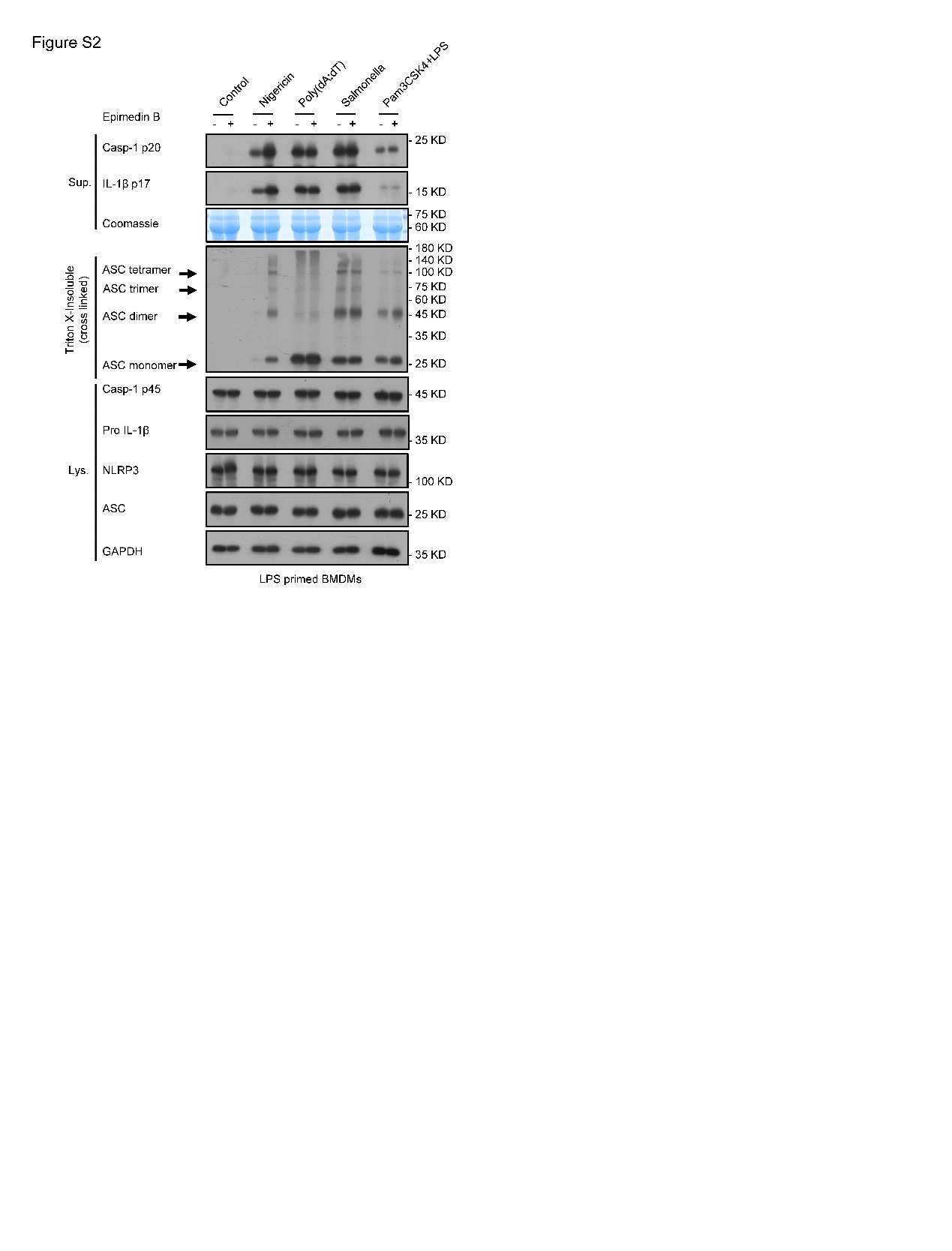


**Supplementary Fig 2 Epimedin B specifically promotes nigericin-induced ASC oligomerization** Western blot analysis of ASC oligomerization from LPS/Pam3CSK4-primed BMDMs subjected to treatment with epimedin B, following which they were stimulated with nigericin, poly (dA:dT), *Salmonella*, or intracellular LPS.


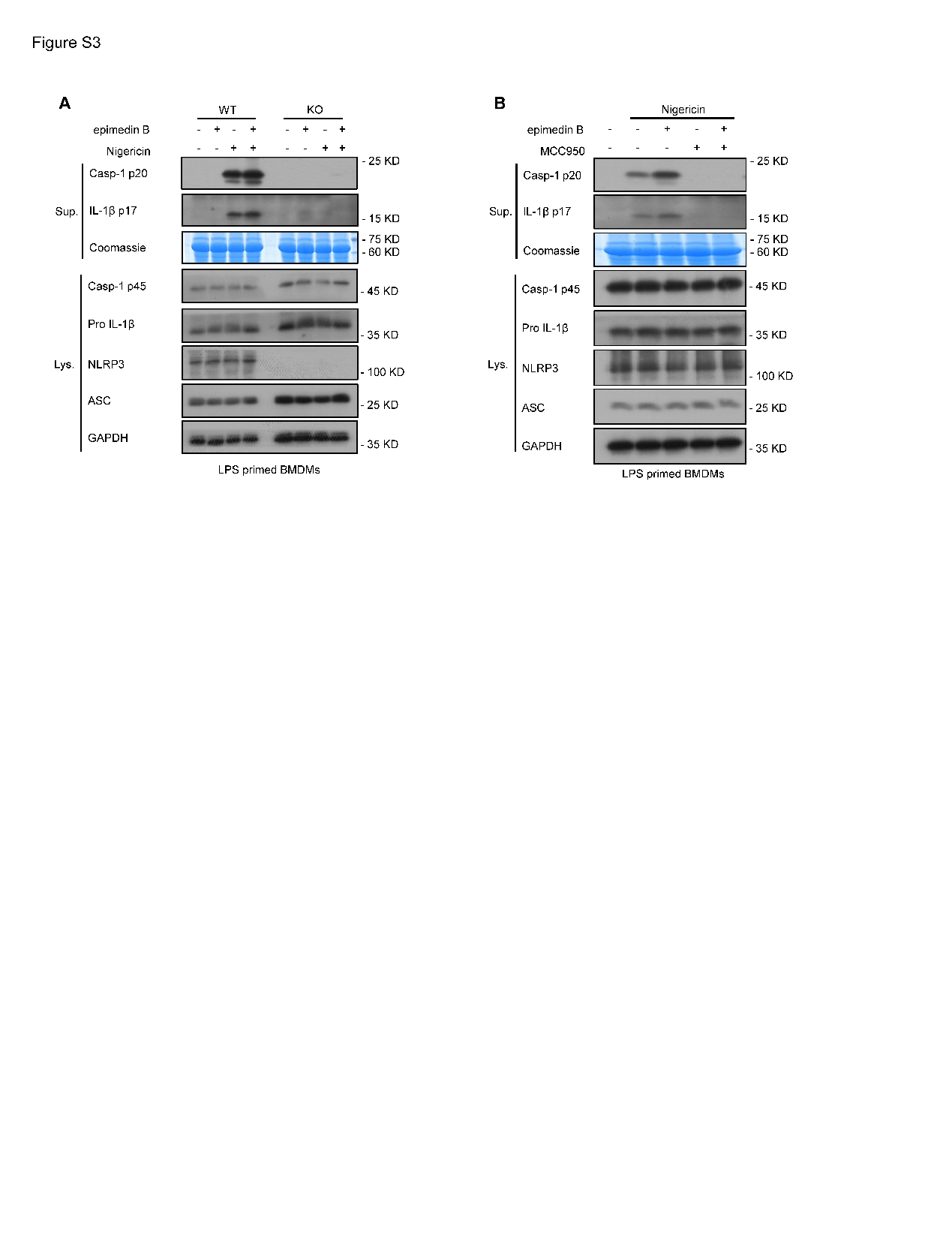


**Supplementary Fig 3 Epimedin B specifically promotes nigericin or ATP-induced NLRP3 activation** (A) Western blot analysis of Sup. and Lys. derived from LPS-primed BMDMs from WT or KO C57BL/6 treatment with epimedin B before nigericin stimulation. (B) Western blot analysis of Sup. and Lys. derived from LPS-primed BMDMs subjected to treatment with epimedin B after treated with MCC950 and then stimulated with nigericin.
